# Adaptation and Validation of the Cognitive and Affective Mindfulness Scale-Revised (CAMS-R) in People Living with HIV in Myanmar

**DOI:** 10.1007/s12671-021-01784-5

**Published:** 2021-11-09

**Authors:** Feifei Huang, Wei-Ti Chen, Cheng-Shi Shiu, Sai Htun Lin, Min San Tun, Thet Wai Nwe, Yin Thet Nu Oo, Htun Nyunt Oo

**Affiliations:** 1 School of Nursing, Fujian Medical University, Fuzhou, China; 2 School of Nursing, University of California Los Angeles, Los Angeles, CA 90095 USA; 3 Department of Social Work, National Taiwan University, Taipei, Taiwan; 4 Advocacy, Human Right & Technical Services Department, Secretariat Office, Myanmar Positive Group (MPG), Yangon, Myanmar; 5 National AIDS Program, Department of Public Health, Ministry of Health and Sports, Naypyitaw, 15011 Myanmar; 6 Department of Medical Research, Deputy Director, Health System Research Division, Yangon, Myanmar

**Keywords:** HIV; Mindful; Myanmar; Psychometrics; Rasch analysis

## Abstract

**Objectives:**

Valid and reliable instruments for the measurement of mindfulness are crucial for people living with HIV. However, there was no Myanmar version of such an instrument.

**Methods:**

We adapted the English version of the 12-item Cognitive and Affective Mindfulness Scale-Revised (CAMS-R) based on standard cross-cultural procedures. By randomly sampling methods, a sample of 248 eligible people living with HIV was contacted from a closed Myanmar Facebook group; 159 PLHIV completed the initial 12-item version of the adapted survey.

**Results:**

Three items were removed due to low item-to-total correlations of the corrected item-total correlation as well as having infit and outfit mean squares outside the range of 0.6 to 1.4. After deleting the 3 items, the three-factor structure was confirmed by confirmatory factor analysis, which indicated good model fit. The resultant 9-item CAMS-R in Myanmar (CAMS-R-M-2) achieved good internal reliability (Cronbach’s *α* of 0.75 to 0.87, and the corrected item-total correlation ranged from 0.44 to 0.81). Construct validity of the scale was demonstrated by significant association with self-reported HIV stigma and social support levels (*r* = 0.63, and − 0.53). In Rasch analysis, the infit and outfit mean squares for each item ranged from 0.49 to 1.24, and the person reliability was 2.17 and the separation index was 0.83.

**Conclusions:**

The 9-item CAMS-R-M-2 with a three-factor structure has good reliability and validity. Higher total scores and subscale score reflected greater mindfulness qualities in people living with HIV in Myanmar.

Advances in medical technologies have changed HIV from a lethal disease to a chronic illness. In addition, multidisciplinary researchers are contributing to improving the wellness of people living with HIV (PLHIV). Even with these scientific advances, however, PLHIV still experience not only physical discomforts but also mental stresses. In particular, the psychosocial outcomes of having the disease have negatively affected patients’ quality of life (QOL) and antiretroviral therapy (ART) adherence (Huynh et al., 2019; Legesse et al. 2019; Relf et al., 2019). Mental health issues of PLHIV include anxiety, depression, disclosure decisions, and perceived stigma, all of which are intertwined with the outcomes of the disease (Chen et al., 2018a, b; Zhang et al., 2017).

To decrease the mental distress of PLHIV, several interventions have been designed and tested, including the Cognitive and Affective Mindfulness Scale (CAMS) intervention (Hunter-Jones et al., 2019; Scott-Sheldon et al., 2019). The overarching goal of mindfulness-based interventions (MBIs) is to increase mindfulness—that is, an individual’s awareness and attention to his or her present moment experiences (Scott-Sheldon et al., 2019). A 2019 study testing the acceptability of a mindfulness-based cognitive therapy intervention for African American women living with HIV showed promising results (Hunter-Jones et al., 2019). According to its authors, this was the first study that used mindfulness-based cognitive therapy with that population. The development of MBIs has been heavily influenced by Buddhist concepts (Wielgosz et al., 2019). However, HIV-related studies conducted in Buddhist-influenced countries have rarely measured the influence of MBIs, other than in the Thai population (Pham et al., 2017).

Myanmar culture is heavily influenced by Buddhism. Similar to neighboring countries, e.g., Cambodia, Laos, and Thailand, Buddhism laid the foundation of Myanmar culture. Nearly 90% of Myanmar’s citizens identify as Buddhists (United Nations Demographic Statistics Database 2017), and nearly all domains of social life are shaped by a Buddhist worldview (de la Perriere 2017). Theravada Buddhism, as an encompassing ideology and civic religion, has provided a unified symbolic system for Myanmar people to interpret and organize their day-to-day lives (Schober, 2011). Buddhists traditionally analyzed patterns in terms of the affective and cognitive “obstacles” to achieving true freedom (Walach et al., 2006). In addition, mindfulness practice, such as meditation, is used to facilitate the recognition of triggers, e.g., sorrow, anxiety, depression, and perceived stigma, and how to avoid them (Walach et al., 2006). To understand how mindfulness impacts care engagement among individuals with HIV in Myanmar, it is critical to contextualize PLHIV’s psychological and behavioral reactions to mindfulness within Buddhist Myanmar culture.

Therefore, the testing of a reliable and valid mindfulness scale is important to further guide the treatment of PLHIV in Myanmar. Currently, there is very limited research on how Myanmar PLHIV live within their cultural interpretations of illness, suffering, and HIV-related issues. However, several instruments have been developed to measure mindfulness. For example, the Freiburg Mindfulness Inventory (FMI; Grossman, 2008) assesses non-judgmental observation of the present moment and how to open oneself to negative experiences. It also applies unique attributes to the mindfulness concept (e.g., verbal description) that are based on elements of dialectical behavior therapy (DBT) that reflect the researchers’ psychiatric background (Baer et al., 2006). For the Cognitive and Affective Mindfulness Scale-Revised (CAMS-R; Feldman et al., 2007), the four major components focusing on mindfulness are (1) the ability to regulate attention, (2) an orientation to present or immediate experience, (3) awareness of experience, and (4) an attitude of acceptance or non-judgment toward experience (Feldman et al., 2007). Another instrument is the Five Facet Mindfulness Questionnaire (FFMQ; Baer et al., 2006), which measures psychometric characteristics.

In this study, we adapted the CAMS for use with the Myanmar population because it captures a multi-faceted conceptualization of mindfulness, is relatively brief, and uses language and a format that does not restrict its use to a specific setting (e.g., mindfulness meditation training; Feldman et al., 2007). CAMS also incorporates four of the five subscales of the Five Facet Mindfulness Questionnaire (Baer et al., 2006), using an abbreviated version of the measure that includes four facets: observing, describing, attention/awareness, and non-judging (Feldman et al., 2007). In addition, the CAMS considers “cognitive, attentional, and behavioral flexibility as components and consequences of mindfulness” (Feldman et al., 2007). Thus, in this study, the aim was to adapt the CAMS-R into a Myanmar version and examine its psychometric properties with both classical test theory (CTT) and the Rasch analysis method among Myanmar PLHIV.

## Method

### Participants

From January 2020 to June 2020, a sample of 248 eligible PLHIV was recruited from a Facebook group list that included more than 10,000 Myanmar people, 90% of whom were PLHIV, by randomly sampling methods; that is, one of every five individuals on the Facebook site roster was contacted. Participants completed a screening questionnaire to ensure they were at least 18 years of age, diagnosed with HIV, able to provide informed consent, and lived within Myanmar.

Of the 248 PLHIV participants, 64.11% (159/248) completed the questionnaires. The mean age of participants was 28.77 years (*SD* = 16.85), and the average years of living with HIV was 7.06 years (*SD* = 6.61). The average recent CD4 count was 678.93 (*SD* = 483.54), and the average viral load was 615.80 (*SD* = 1058.55). Table 1 presents details of the sociodemographic characteristics of the participants.

**Table 1:** Sociodemographic characteristics of the participants (*N* = 159)

Variables	*N* (%)
Gender	
Male	99 (62.30%)
Female	58 (36.50%)
Transgender	2 (1.3%)
Ethnicity^a^	
Bamar	123 (77.8%)
Chin	2 (1.3%)
Kachin	2 (1.3%)
Kayin	6 (3.8%)
Kayah	1 (0.6%)
Mon	9 (5.7%)
Rakhine	4 (2.5%)
Shan	4 (2.5%)
Others*	7 (4.4%)
Marital status^a^	
Married or steady partner	65 (41.1%)
Widowed	19 (12.0%)
Separated	6 (3.8%)
Divorced	11 (7.0%)
Single, never married	57 (36.1%)
Educational level	
Middle school graduation	19 (11.9%)
High school graduation	68 (42.8%)
Professional (vocational) training school graduation	1 (0.6%)
Some college but no degree	24 (15.1%)
College graduation	44 (27.7%)
Post college graduate	3 (1.9%)
Employment status^a^	
No	32 (20.2%)
Part time	33 (20.9%)
Full time	93 (58.9%)
Health insurance^a^	
Not enough	131 (84.0%)
Just enough	25 (16.0%)

^*^Palaung, Islam, Tamil

^a^Missing data

### Procedures

This cross-sectional descriptive study was approved by the relevant institutional review boards (#18–001,769), and written informed consent was obtained from the participants. We culturally adapted the CAMS for individuals with HIV in Myanmar and examined the psychometric properties of the scale, which were in adherence to the **CO**nsensus-based **S**tandards for the selection of health status **M**easurement **IN**struments (COSMIN) checklist (Mokkink et al., 2010a, 2010b). If participants agreed to participate in this study and were able to provide informed consent, a link to the survey was sent to them. All information was collected online through the Research Electronic Data Capture (REDCap) system, a web-based survey tool that was supported through the involved research institution (Harris et al., 2009, 2019). After completing the survey, participants were reimbursed for their participation and time.

### Measures

Participants completed the 30-min REDCap survey, which consisted of standardized measures to assess demographics, CAMS, and the Center for Epidemiological Studies Depression Scale (CES-D; Radloff, 1977; the overall Cronbach *α* in this sample was 0.83).

#### Demographics

Participant age, gender, marital status, ethnicity, education level, employment status, years of living with HIV, type of antiretroviral therapy, and recent CD4 and viral load were collected.

Cognitive and Affective Mindfulness Scale-Revised (CAMS-R; Feldman et al., 2007).

This 12-item scale measures the mindfulness experienced by individuals in four dimensions: attention, present focus, awareness, and acceptance (e.g., “It is easy for me to concentrate on what I am doing,” “I am easily distracted”). All the items were rated using a 4-point Likert scale (1 = *rarely/not at all* to 4 = *almost always*). After reversing the scores of items 2, 6, and 7, higher total scores reflected greater mindfulness qualities. In this study, this scale was used to measure the mindfulness experienced by PLHIV in Myanmar and was adapted in the following three phases.

We adapted Brislin’s translation model and applied it to cross-cultural translation, which included using translation, back-translation, comparison, and linguistic adaptation (Brislin, 1970; Jones et al., 2001). The 12-item CAMS-R was translated independently from English into Myanmar by a bilingual physician who was providing HIV care in Myanmar and had native proficiency in Burmese and English. Then, a bilingual researcher (Myanmar-English) who was blinded to the CAMS-R English items back-translated the Myanmar version into English. Later, one member (FFH, Ph.D. in nursing major) of the research team compared the back-translated English version with the original English scale. No item was found to be discrepant. Then, a pilot test was distributed to 10 PLHIV in Myanmar to evaluate the items’ fluency, readability, and comprehensibility. We made appropriate modifications according to their feedback. After this process, the final Myanmar version of the 12-item CAMS-R was ready for validation (CAMS-R-M-1). The CAMS-R-M-1 was completed by 248 PLHIV participants, and the reliability and validity of the scale were examined by the CTT and Rasch analysis (Leung et al., 2014).

### Data Analyses

Data analyses were conducted using SPSS 23.0 (IBM, Chicago, IL, USA) and Mplus 6.1 (Muthén & Muthén, Los Angeles, CA, USA). Also, the Rasch analysis was conducted using WINSTEPS 3.75.0 (Chicago, IL, USA). Missing data of the CAMS-R-M-1 used the mean imputation calculation. *p* < 0.05 was considered significant.

- The sociodemographic characteristics of the participants were analyzed by statistical description, that is, continuous variables are expressed as means and standard deviations (SDs), and categorical variables are expressed as proportions or percentages.
- Item retention analysis: We deleted the item if it met the following criteria of CTT and Rasch analysis: (1) the corrected item-total correlation was not statistically significant (*p* < 0.05) and (2) infit and outfit mean squares were outside the range of 0.6 to 1.4. After items were deleted, we increased the *α* coefficient for the overall scale (Huang et al., 2017; Xu et al., 2018).
- Structural validity: We used confirmatory factor analysis (CFA) to examine the best fitting model of the scale using the method of maximum likelihood. The model’s goodness of fit was evaluated using absolute and relative indices (Huang et al., 2017; Xu et al., 2018), including normed *χ*^2^ (*χ*^2^/df) between 1.0 and 3.0, Root Mean Square Error of Approximation (RMSEA; < 0.08), Comparative Fit Index (CFI), Tucker-Lewis Index (TLI), and Normed Fit Index (NFI) > 0.9.
  In the Rasch analysis, we first examined the unidimensionality assumptions by the first contrast of the residual, which generally should not be above 2 (Leung et al., 2014). Then, we used the unrestricted Partial Credit Rasch (PCR) model to assess person separation reliability, person separation index, person fit statistics, and test information function (TIF; Johnson et al., 2011; Xu et al., 2018). The PCR is an item response theory model for polytomous items with ordered categories (Masters, 1982). Pearson fit statistics included infit and outfit mean squares, as well as difficult (location) for individual items. TIF was produced from the sum of each item and information curve in each subscale. Then, the depicted items with the levels of Ө could most precisely and reliably gather the necessary information (Baker, 2001). Items were tested for the differential item functioning (DIF) across gender (male, female, and transgender). Finally, ordinal-to-interval transformation scores were calculated for users to transform ordinal data to an interval-level scale.
- Construct validity: We estimated the construct validity of the CAMS-R by Pearson’s correlations with the expected significantly negative correlation of the CES-D.
- Inter-item consistency was estimated by Cronbach’s *α*, and reliability was estimated by corrected item-total correlation, and Mean Inter-Item Correlations (MIIC).
- Floor/ceiling effect: Floor effects were evaluated by examining the percentage of the respondents who achieved the lowest possible scores. Ceiling effects were evaluated by examining the percentage of respondents who reached the highest possible score.

## Results

As shown in Table 2, according to the criteria of item retention, three items (I-2, I-6, and I-7) were removed due to the insignificance of corrected item-total correlation, as well as having infit and outfit mean squares outside the range of 0.6 to 1.4. After the items were deleted, the alpha coefficient for the overall scale was increased. Thus, the final 9-item CAMS-R-M-2 was formed (see Table 3).

**Table 2:** Item and factor analysis of the scale

Item	Corrected item-total correlation	Infit MNSQ	Outfit MNSQ	Cronbach’s *α* after removing the item*	Item retention or not
I-1	0.62	0.78	0.76	0.71	Yes
I-2	− 0.19**	1.88	2.04	0.80	No
I-3	0.59	0.80	0.81	0.72	Yes
I-4	0.59	0.91	0.89	0.71	Yes
I-5	0.47	0.87	1.01	0.73	Yes
I-6	− 0.11**	1.53	1.61	0.79	No
I-7	0.09**	1.13	1.22	0.77	No
I-8	0.32	1.12	1.10	0.75	Yes
I-9	0.39	1.04	1.04	0.74	Yes
I-10	0.77	0.52	0.52	0.69	Yes
I-11	0.65	0.76	0.75	0.71	Yes
I-12	0.62	0.66	0.70	0.71	Yes

^*^Before item reduction, the overall Cronbach *α* = 0.756

^**^p ≤ 0.05

**Table 3:**
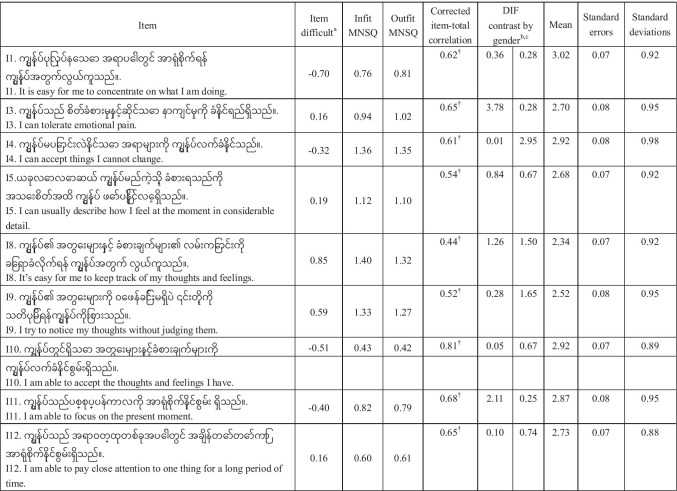
The difficult, infit, outfit MNSQ, and corrected item-total correlation of 9 items

^a^Measured in logit; positive item logit indicates that the item requires a lower visual ability than the mean of the items and is an easier item; while a negative item logit indicates that the item requires a higher visual ability than the mean of the items and is a more difficult item

^†^*p* ≤ 0.05; MNSQ mean square

The DIF contrast by gender in the following order:

^b^Male compared with female

^c^Male compared with transgender

**Fig. 1:**
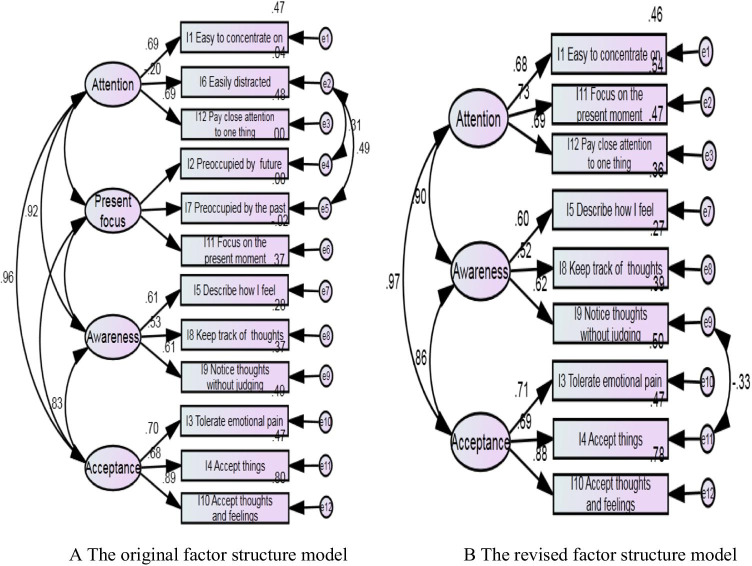
**A** The original factor structure model. **B** The revised factor structure model

The original CAMS-R has a four-factor structure, that is, the factors of attention (I-1, I-6, I-12), present focus (I-2, I-7, I-11), awareness (I-5, I-8, I-9), and acceptance (I-3, I-4, I-10; Feldman et al., 2007). In this study, after three items (I-2, I-6, and I-7) were removed, the factor of present focus was left with one item (I-11: *I am able to focus on the present moment*). Therefore, the I-11 item was attributed to the factor of attention after research team discussion. We then conducted a CFA to examine and compare the factor structure proposed by the original CAMS-R and the revised factor structure of CAMS-R-M-2. As shown in Fig. 1, the four-factor structure in the original CAMS-R was not confirmed due to the insignificant contribution of the present-focus factor (*χ*^2^ (46) = 2.61, *p* = 0.01, RMSEA = 0.10, CFI = 0.90, and TLI = 0.85), while the three-factor structure of CAMS-R-M-2 was confirmed (*χ*^2^ (23) = 2.163, *p* = 0.01, RMSEA = 0.08, CFI = 0.95, and TLI = 0.93), and labeled as (a) Attention, (b) Awareness, and (c) Acceptance.

**Fig. 2:**
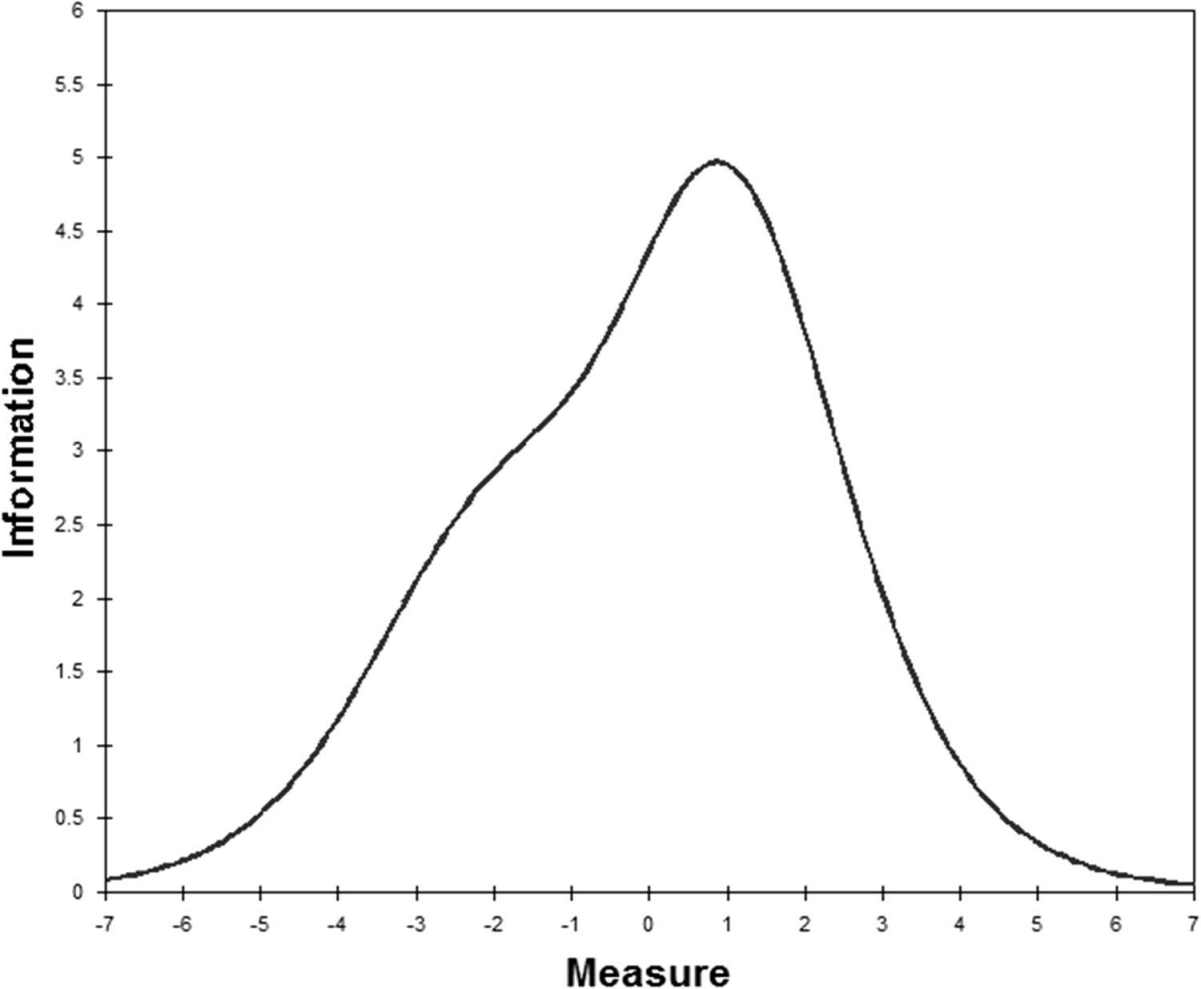
Test information function for the 9-item Myanmar version of the Cognitive and Affective Mindfulness Scale-Revised

**Fig. 3:**
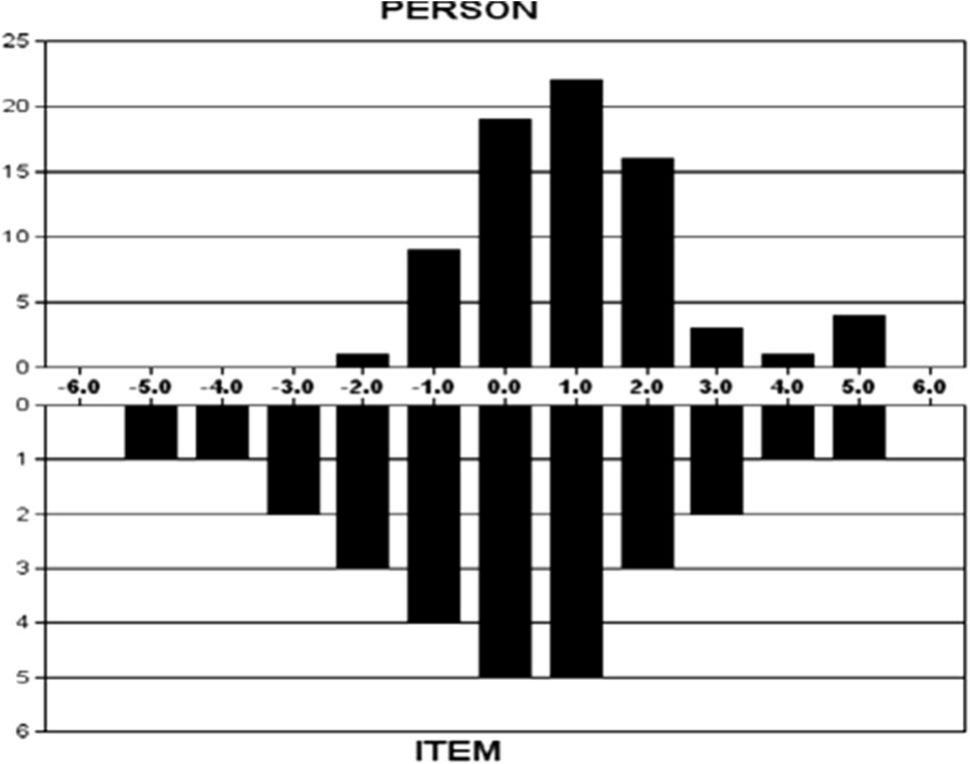
The person-item threshold distribution plot

In the Rasch analysis, the unidimensionality assumption of scale was supported by the first contrast of the residual, which was 1.8 (less than 2). As shown in Table 3, the infit and outfit mean squares for each item ranged from 0.42 to 1.40. Differential item functioning was not found when evaluated by gender. We also found the item reliability (0.93), item separation index (3.64), person reliability (2.17), and the person separation index (0.83) in the analysis. Regarding the TIFs, both subscales gathered information most precisely when Ө ranged from 0 to 2.0 (see Fig. 2). The person-item threshold distribution plot is shown in Fig. 3. The raw score means, standard deviations, and standard errors for each item are also shown in Table 3. The ordinal-to-interval conversion table is presented in Table 4.

**Table 4:** Converting from ordinal- to interval-level scores for the total score of the CAMS-R-M

Raw score	Interval-level score	Raw score	Interval-level score
0	− 4.14	11	− 0.43
1	− 3.81	12	− 0.10
2	− 3.47	13	0.24
3	− 3.13	14	0.58
4	− 2.80	15	0.91
5	− 2.46	16	1.25
6	− 2.12	17	1.59
7	− 1.78	18	1.93
8	− 1.45	19	2.26
9	− 1.11	20	2.60
10	− -0.77		

The convergent validity for the CAMS-R-M-2 (version 2) was confirmed with negative correlation with the CES-D (*r* =  − 0.70, *p* < 0.001). Furthermore, the three subscales of the CAMS-R-M-2 also have negative correlations with the CES-D (*r* =  − 0.625, − 0.639, − 0.647, *p* < 0.001).

The Cronbach alpha was 0.87 for the CAMS-R-M-2 (0.61 for the Awareness factor, 0.75 for the Attention factor, and 0.81 for the Acceptance factor) and 0.75 for the CAMS-R. The corrected item-total correlation ranged from 0.44 to 0.81 (*p* < 0.05). The MIIC was 0.43 for the CAMS-R-M-2 (0.34 for the Awareness factor, 0.50 for the Attention factor, and 0.56 for the Acceptance factor).

Of the total number of participants, 0.06% (1/159) and 3.14% (5/159) achieved the lowest possible score (9) and the highest possible score on the scale (36), respectively. The lowest and highest possible scores were both below 15%, indicating that there were no floor or ceiling effects of the CAMS-R-M-2 (Terwee et al., 2007).

## Discussion

This paper concerns an initial scale validation study focusing on a mindfulness scale administered to an HIV-infected population, particularly in the Myanmar Buddhist context. This mindfulness scale validation comprised a multiphase process to ensure the rigorousness of the scale validation. The psychometric evaluation presented in this paper provides satisfactory cross-cultural, structural, and construct validities, as well as robust internal consistency reliability. Floor and ceiling effects were not present. Therefore, the final 9-item CAMS-R-M-2 can serve as a valid and reliable scale to quantify the cognitive and affective mindfulness in PLHIV in Myanmar.

The factor analytic strategies used in CTT yielded a clear three-factor structure for the 9-item CAMS-R-M-2. This finding shows that mindfulness differs as a construct across cultures (Ramirez-Garcia et al., 2019; Scott-Sheldon et al., 2019; Wielgosz et al., 2019). In this study, the CAMS-R-M-2 was adapted from Feldman’s Cognitive and Affective Mindfulness Scale, which measures a person’s ability to regulate attention, orientation to present or immediate experience, awareness of experience, and an attitude of acceptance or non-judgment toward experience (Feldman et al., 2007, 2011). What’s more, the higher total scores reflected greater mindfulness qualities in PLHWA.

In contrast to Feldman’s CAMS-R, only 9 items of the CAMS scale were left in the Myanmar version, and item I-11 (*I am able to focus on the present moment*) was attributed to the factor of attention, which might further confirm the influence of traditional Buddhist concepts among PLHWA in Myanmar. As these three removed items asked PLWHA about the future (I-2) and the past (I-7), and whether the respondent was easily distracted (I-6), these concepts did not align with Buddhist doctrine and the fact these people grew up in a country with a heavy focus on “living in the present.” It might also be because of the unstable political history of Myanmar, which includes unending civil war, and military rule, which leads many people in Myanmar to not want to refresh their memory of the civil unrest and to not dare to plan for their future, and, thus, just live in the present (Walton, 2016). Therefore, only one question in the “present focus” factor (I-11: *I am able to focus on the present moment*) was significant in this study analysis.

Also, the remaining items were found to have a similar factor structure to the scale as previously presented in an American context (Chen et al., 2018a, b). This finding indicates that this scale can be used to measure the past and present experiences of mindfulness, as well as attention among PLHWA in Myanmar. In addition, the reduced items further suggested that part of the CAMS scale was redundant to the Myanmar PLHWA.

In the traditional CTT method, the structural validity of the CAMS-R-M-2 was confirmed by the Rasch analysis (Kalmbach et al., 2020; Lima et al., 2019). The scale validation of this study supports the category rating scale of the CAMS-R-M-2 and that it is free of DIF by gender. The combination of a good person separation index (> 2) and person reliability (> 0.8) suggests that the CAMS-R-M-2 has good measurement precision and is sensitive enough to distinguish both participants who have highly effective and less effective mindfulness experiences (Kemper, 2017; Russell et al., 2018; Shaffer et al., 2016). As shown in Table 4, the ordinal-to-interval conversion tables for the CAMS-R-M-2 permit transformation of ordinal responses into interval-level data to increase the accuracy of measurement (Leung et al., 2014). There are no ceiling or flow effects if transformation table is used, which support robustness of the transformed scale with the target population. As shown in Fig. 3, the person-item threshold distribution map shows that participants are distributed in a similar fashion to the items, which is indicative that the items measure mindfulness along the construct from “rarely/not at all” to “almost always.” Regarding the TIF, when represented graphically, high TIF values are associated with low standard errors of measurement and, thus, can indicate precision (Huang et al., 2017). The most precise information provided by the TIF for the CAMS-R-M-2 show the precise and reliable measure of the middle levels of this Myanmar version of the Cognitive and Affective Mindfulness Scale.

Similar to previous studies (Scott-Sheldon et al., 2019; Waldron 2019; Wielgosz et al., 2019), the construct validity of the scale was supported, as there were significantly positive correlations with self-reported depressive symptomology. In addition, the Cronbach *α* of more than 0.7 indicated that the CAMS-R-M-2 had satisfactory internal consistency reliability, which is similar to another study in the USA (Britton et al., 2014). In this study, we found that the overall alpha of 0.87 was higher than any individual (revised) factor alpha. The possible reason for this is that Cronbach’s alpha is a function of the number of items in a test, the average covariance between pairs of items, and the variance of the total score (Huang et al., 2017).

Evidence has indicated that mindfulness practice can enhance quality of life (Balthip et al., 2013; Grant, 2014). In resource-limited countries where religious practice plays an important role in life, such as Myanmar, one of the ways to enhancing quality of life is mindfulness practice (Balthip et al., 2013; Bharat, 2011; Bhochhibhoya et al., 2018). The psychometric properties presented in this paper suggest that the 9-item CAMS-R-M-2 can accurately measure attention, orientation to present and immediate experience, awareness of experience, and an attitude of acceptance of experience that can improve the peace of mind of PLHIV in Myanmar. This scale can also facilitate the development of culturally sensitive interventions and evaluations of the effects of future interventions in countries where the practice of mindfulness occurs. In particular, for cultures heavily influenced by the Buddhist doctrine to “live in the present,” future intervention designs could lead PLHIV to seek the meaning of their lives in the present moment and relieve stress through meditation. Future research in more representative samples is needed to further examine the screening utility of this scale. It will also be important to determine the cutoff value for the CAMS-R-M-2 and compare the effectiveness of mindfulness practice by PLHIV globally.

### Limitations and Future Research

This study has several limitations. First, this sample size was relatively small and some psychometric characteristics of the CAMS-R-M-2 should be assessed further, such as test–retest reliability. Second, the sensitivity of the CAMS-R-M-2 was not assessed. Third, it is not clear how possible increased stress due to COVID-19 may have impacted the participants and, therefore, the data collected. Therefore, future longitudinal experimental studies are warranted. A further refinement of the scale based on the testing of the scale with a larger representative sample will produce more stable parameter estimation and robust results.

## Data Availability

Data are available upon request.

## Declarations

### Disclaimer

The contents of this article are solely the views of the authors and do not represent the official views of the National Institutes of Health.
